# Draft Genome Sequence of *Candida saopaulonensis* from a Very Premature Infant with Sepsis

**DOI:** 10.1007/s11046-024-00838-1

**Published:** 2024-04-15

**Authors:** Ya-Ting Ning, Rong-Chen Dai, Zheng-Yu Luo, Meng Xiao, Yingchun Xu, Qun Yan, Li Zhang

**Affiliations:** 1 Department of Laboratory Medicine, State Key Laboratory of Complex Severe and Rare Diseases, Peking Union Medical College Hospital, Chinese Academy of Medical Sciences, Beijing, 100730 China; 2 Beijing Key Laboratory for Mechanisms Research and Precision Diagnosis of Invasive Fungal Diseases, Beijing, 100730 China; 3 Department of Clinical Laboratory, Xiangya Hospital, Central South University, Changsha, 410008 China

**Keywords:** *Candida saopaulonensis*, Clinical case, Whole genome sequencing, Genome assembly, Annotation, Fungi

## Abstract

The rare fungus *Candida saopaulonensis* has never been reported to be associated with human infection. We report the draft genome sequence of the first clinical isolate of *C. saopaulonensis,* which was isolated from a very premature infant with sepsis. This is the first genome assembly reaching the near-complete chromosomal level with structural annotation for this species, opening up avenues for exploring evolutionary patterns and genetic mechanisms of pathogenesis.

**Supplementary Information:**

The online version contains supplementary material available at 10.1007/s11046-024-00838-1.

The rare fungus *Candida saopaulonensis* belongs to the Metschnikowiaceae clade, and is genetically most closely related to *Candida picinguabensis*. These two species are similar in morphology and physiology, but nucleotide differences, with 18 substitutions and three gaps in the D1/D2 domain of the large-subunit rDNA, warranted their classification as distinct species [1]. *C. saopaulonensis* was first isolated from water in the flower bracts in southeastern Brazil in 2000, and then found on corn plants in Thailand in 2016 [1, 2]. Human infections caused by this species have not been previously reported.

A 4-week-old male very premature infant, with gestational age of 29 weeks and birth weight of 780 g, was admitted for bronchopulmonary dysplasia. *C. saopaulonensis* was recovered from blood cultures obtained aseptically from the catheter hub on 3 June, 2019. The subculture demonstrated the phenotype of smooth globose colonies on Sabouraud dextrose medium (Thermoscientific, USA), and of ‘gray purple’ on Candida chromogenic medium (CHROMagar, France). Optical microscopy showed small round or ovoid cells, appearing singly or in the budding state. Formation of hyphae or pseudohyphae was not observed (Supplementary Fig. 1). Antifungal susceptibilities were tested using YeastOne Sensititre (Thermo Fisher, USA), and the minimum-inhibitory concentrations were as follows: fluconazole 0.12 mg/L, itraconazole 0.06 mg/L, voriconazole 0.008 mg/L, posaconazole ≤ 0.008 mg/L, micafungin 0.25 mg/L, anidulafungin 1 mg/L, caspofungin 0.5 mg/L, flucytosine ≤ 0.06 mg/L, and amphotericin B ≤ 0.12 mg/L.

This isolate (19XY460) was misidentified by Autof ms 1000 (Autobio Diagnostics Co., Ltd, China) as *C. parapsilosis* sensu stricto*.* Whole-genome sequencing of the isolate was carried out using Illumina MiSeq PE150 system, and data quality control and single nucleotide polymorphism (SNP) calling were performed as previously described [3]. Compared to the reference genome NRRL Y-27815 (GenBank assembly accession: GCA_030582915.1), a total of 100,171 SNP positions and 8455 insertion-deletion positions were identified in the genome of 19XY460. Alignment of the internal transcribed spacer (ITS) regions showed a 100% nucleotide identity of the sequence from isolate 19XY460 with that from *C. saopaulonensis* strain C6A (GenBank Accession No. KX781276.1), while it showed a 98.9% (370 bp /374 bp) identity with *C. saopaulonensis* type strain NRRL Y-27815; the latter comparison identified four differences, C281A, C282A, T283A, and C285T mutations, in the ITS regions of 19XY460 and NRRL Y-27815. There were no large-scale amplifications in this genome (Supplementary Fig. 2A). Ploidy analysis, visualized using the frequency of the non-reference allele across cumulative from heterozygous biallelic SNPs throughout all chromosomes [4], revealed that the isolate 19XY460 was haploid, characterized by almost entirely homozygous SNP sites (Supplementary Fig. 2B).

Long-read sequencing was performed using the PacBio Sequel II platform, and the data were assembled to the chromosomal level using Canu v2.2 [5]. The assembly was polished with next-generation sequencing data by Pilon v1.23 [6], and BUSCO v5.2.2 was used for quality control of assembly completeness [7]. The genome assembly resulted in eight scaffolds, with a total length of 12,068,158 bp (11.51 Mb), which was quite similar to the NRRL Y-27815 genome (12 Mb). The completeness of this genome reached 97.85%. GC content was 44.27%, and N50 was improved from 547,923 bp (NRRL Y-27815) to 2,019,073 bp (19XY460). The results of average nucleotide identity, determined with pyani tool, indicated that the genome of isolate 19XY460 had a 98.95% similarity with *C. saopaulonensis* strain NRRL Y-27815, and 95.64% similarity with the closely related fungus *C. picinguabensis* strain NRRL Y-27814 (Supplementary Fig. 2C&D). Structural annotation of the genome was conducted using AUGUSTUS v3.3.2 [8], then assessed using BUSCO v5.2.2 [7]. A total of 5006 genes were annotated on the chromosomes of 19XY460, achieving an annotation completeness of 96.17%. Additionally, the mitochondrial genome was assembled using GetOrganelle v1.7.7.0 [9], resulting in a circular sequence with a length of 41,901 bp and a GC content of 30.77%.

To the best of our knowledge, this is the first report of clinically relevant case caused by the rare fungal species *C. saopaulonensis.* We also describe the first genome assembly data of this species to the near-complete chromosomal level, complemented by structural annotation. As fungal diseases become more prevalent, this invaluable resource opens up avenues to delve into the evolutionary patterns and genetic mechanisms of pathogenesis of a potential new pathogen, *C. saopaulonensis*.

### Supplementary Information

Below is the link to the electronic supplementary material.

Supplementary file1 (DOCX 877 KB)

## Data Availability

The isolate 19XY460 has been deposited at China Center of Industrial Culture Collection under the accession number CICC 33624 (http://www.china-cicc.org/cicc/detail2/?sid=14028). All sequencing data, assembly and annotation results have been deposited in the NCBI database (Biosample Number SAMN37476331, BioProject Number PRJNA1019270, Assembly Accession Number GCA_035610405.1, and ITS Accession Number PP123913.1). Data analysis and writing of article were in compliance with the MycopathologiaGENOMES quality assurance checklist [10].
