# Supplementary material for: Draft Genome Sequence of Candida saopaulonensis from a Very Premature Infant with Sepsis
Source: Mycopathologia. 2024 Apr 15;189(3):32. doi: 10.1007/s11046-024-00838-1 (PMC11018655; doi:10.1007/s11046-024-00838-1)
Supplement: Supplementary file 1 — Supplementary file1 (DOCX 877 KB) [file 11046_2024_838_MOESM1_ESM.docx]

**Supplementary Data**


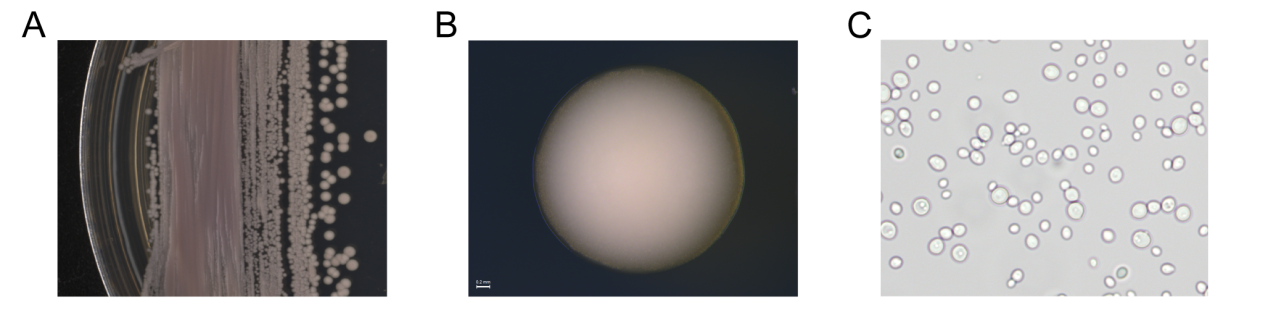


**Supplementary Figure 1.** Colony and cell morphology of *C. saopaulonensis* after 48 h incubation at 30°C in ambient air. (A) Gray/purple globose colonies of *C. saopaulonensis* on Candida chromogenic medium; (B) Smooth white globose colonies of *C. saopaulonensis* on Sabouraud dextrose agar; (C) Small round or ovoid cells of *C. saopaulonensis* with optical microscope, appearing in single or in budding state. Formation of hyphae or pseudohyphae was not observed.


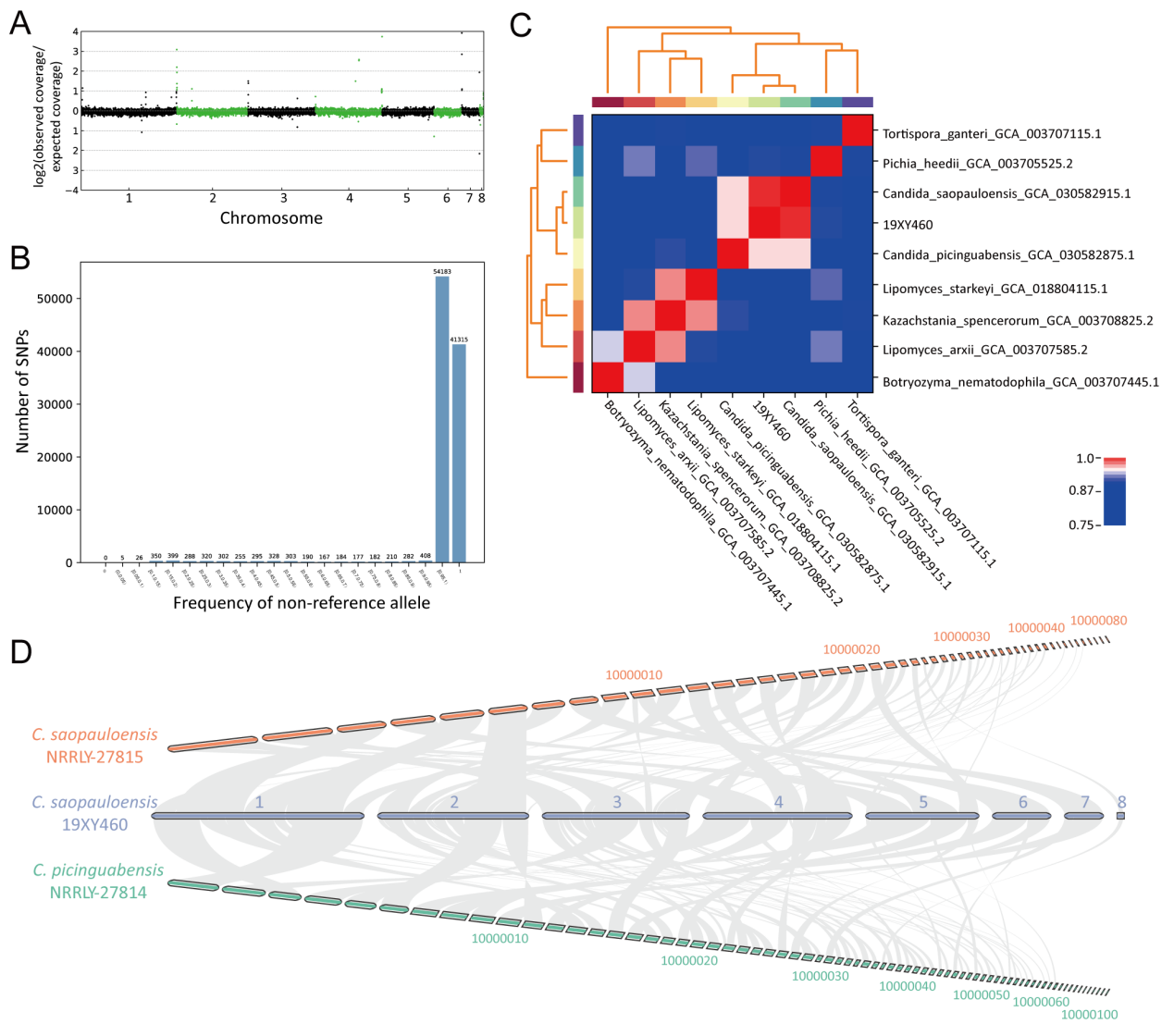


**Supplementary Figure 2.** Genomic characteristics of *C. saopaulonensis* isolate 19XY460. (A) Analysis of large-scale amplifications in genome of 19XY460, and each spot represents the sequence depth for 1000 bp windows across the genome; (B) Ploidy analysis based on frequency of the non-reference allele for all heterozygous biallelic SNPs across the genome of 19XY460; (C) Heatmap of average nucleotide identity analysis results; (D) The genome collinearity among 19XY460 with *C. saopaulonensis* strain NRRL Y-27815 and *C. picinguabensis* strain NRRL Y-27814.
